# Efficacy of graded activity versus supervised exercises in patients with chronic non-specific low back pain: protocol of a randomised controlled trial

**DOI:** 10.1186/1471-2474-14-36

**Published:** 2013-01-21

**Authors:** Mauricio Oliveira Magalhaes, Fábio Jorge Renovato França, Thomaz Nogueira Burke, Luiz Armando Vidal Ramos, Ana Paula de Moura Campos Carvalho e Silva, Gabriel Peixoto Leao Almeida, Susan Lee King Yuan, Amélia Pasqual Marques

**Affiliations:** 1 Department of Physical Therapy, Communication Science & Disorders, Occupational Therapy, University of São Paulo, São Paulo, Brazil

## Abstract

**Background:**

Low back pain is a relevant public health problem, being an important cause of work absenteeism worldwide, as well as affecting the quality of life of sufferers and their individual functional performances. Supervised active physical routines and of cognitive-behavioral therapies are recommended for the treatment of chronic Low back pain, although evidence to support the effectiveness of different techniques is missing. Accordingly, the aim of this study is to contrast the effectiveness of two types of exercises, graded activity or supervised, in decreasing symptoms of chronic low back pain.

**Methods/design:**

Sample will consist of 66 patients, blindly allocated into one of two groups: 1) Graded activity which, based on an operant approach, will use time-contingent methods aiming to increase participants’ activity levels; 2) Supervised exercise, where participants will be trained for strengthening, stretching, and motor control targeting different muscle groups. Interventions will last one hour, and will happen twice a week for 6 weeks. Outcomes (pain, disability, quality of life, global perceived effect, return to work, physical activity, physical capacity, and kinesiophobia) will be assessed at baseline, at treatment end, and three and six months after treatment end. Data collection will be conducted by an investigator blinded to treatment allocation.

**Discussion:**

This project describes the randomisation method that will be used to compare the effectiveness of two different treatments for chronic low back pain: graded activity and supervised exercises. Since optimal approach for patients with chronic back pain have yet not been defined based on evidence, good quality studies on the subject are necessary.

**Trial registration:**

NCT01719276

## Background

Low back pain (LBP) is a relevant public health problem, being an important cause of work absenteeism worldwide [1-3], as well as affecting sufferers’ quality of life [1] and individual functional performances [1]. Non-specific chronic LBP (cLBP), which does not have a well-defined etiology and presents pain for at least 12 consecutive weeks, represents up to 95% of the cases of LBP [1]. The annual direct costs of cLBP in the United States range from 12.2 to 90.6 billions of dollars, and represent only 14.5% of the total costs of this health condition [4]. Life-time prevalence of cLBP ranges from 11 to 84%; 1-year prevalence ranges from 22% to 65%, and point-prevalence from 12% to 33% [5].

The European Guidelines recommends the use of supervised active exercises, manipulation/mobilization, Back Schools, multidisciplinary approaches and cognitive-behavioral therapies for patients with cLBP [1]. Evidence suggests that supervised exercise and cognitive behavioral therapies improve pain and reduce functional disability [6].

Strengthening exercises for abdominal and trunk muscles, motor control exercises for lumbar multifidus (LM) and transversus abdominis (TrA) and stretching exercises for trunk and lower limbs show some evidence of improvement of pain and functional disability in individuals with cLBP [1,7,8]. Strengthening exercises of abdominal and trunk muscles are based on the known association between weakness of the trunk and abdomen muscles and low back pain [9-14]. Weakness is a consequence of sedentary life, and is associated to paravertebral muscle hypotrophy [14] and changes in motor control [15]. Furthermore, deep muscles of the abdomen and trunk such as the TrA and LM are also affected in patients with cLBP [16]. Some studies have focused on the individual use of muscle stretching and strengthening or motor control in cLBP [17,18]. However, Macedo and colleagues [19], on their systematic review, recommend motor control exercises associated with other types of exercise.

Cognitive behavioral therapy uses brief interventions and counseling strategies in order to facilitate behavioral changes [20], by modifying negative attitudes and beliefs [21]. The “Back Book” [22] may be used as a good educational support, since it offers evidence-based information that is consistent with biopsychosocial models. Cognitive-behavioral programs that showed some evidence for use in patients with cLBP include the Back Skills Training program (BeST) [21,23], Brief Intervention (BI) [24,25] and the Graded Activity [26].

The Graded Activity program, which was initially developed by Lindstrõm *et al.*[27], recommends the use of an individualized and submaximal exercise program, with educational support in order to enhance self-trust and tolerance to effort. Although it has been suggested that graded activity is effective in decreasing pain and functional disability in cLBP [26,28], van der Giessen (2012), in a systematic review [29], concluded that there is insufficient evidence on the effects of graded activity in pain, disability and return to work in patients with non-specific cLBP. Furthermore, the *Clinical Practice Guidelines Linked to the International Classification of Functioning*, *Disability*, *and Health from the Orthopaedic Section of the American Physical Therapy Association*[3] states that effectiveness of cognitive behavioral therapy in cLBP is moderate.

The literature is not clear regarding which exercise programs are most effective for patients with cLBP; therefore, more randomised controlled trials are necessary to clarify these questions. Moreover, little is known about the effect of graded activity compared with supervised exercise program (strengthening, stretching and motor control) in patients with non-specific cLBP.

### Study aim

The aim of this paper is to report the study protocol used to investigate the effect of two types of exercise program in reducing the symptoms of non-specific cLBP.

## Methods and design

The study will be a randomised controlled trial comparing graded activity with a supervised exercise program in patients with non-specific cLBP. Each treatment program will consist of 12 individually supervised 1-hour sessions over a six-week period.

### Enrollment and eligibility criteria

A total of 66 patients will be recruited at the Rehabilitation Center of Taboao da Serra, Brazil.

#### Inclusion criteria

· Non-specific cLBP;

· Age between 18 and 65 years;

· A minimum pain intensity score of three in the 11-point Pain Numerical Rating Scale [24].

#### Exclusion criteria

· Known or suspected serious spinal pathology (fractures, tumors, inflammatory or infective diseases of the spine);

· Nerve root compromise;

· Comorbid health conditions that would prevent active participation in the exercise programs;

· Pregnancy;

· Cardio-respiratory illnesses.

In order to ensure patients’ safe participation in the study, the *Physical Activity Readiness Questionnaire* (PAR-Q) will be used [30]. Those answering “yes” to any of the questions will be excluded.

### Procedures

All measurements will be conducted by a physical therapist blinded to patients’ groups. The investigator will confirm the eligibility criteria, as well as obtain demographic (civil status, education, tabagism and use of medication) and anthropometric data (age, height, weight and body mass index). Past treatments and use of medication for cLBP will also be recorded. The investigator will also assess primary (pain severity and functional disability) and secondary outcomes (quality of life, global perceived effect, return to work, physical activity, physical capacity and kinesiophobia). Patients will be assessed at baseline, immediately after the treatment and at three and six-month follow-up.

### Randomisation procedures

**Figure 1:**
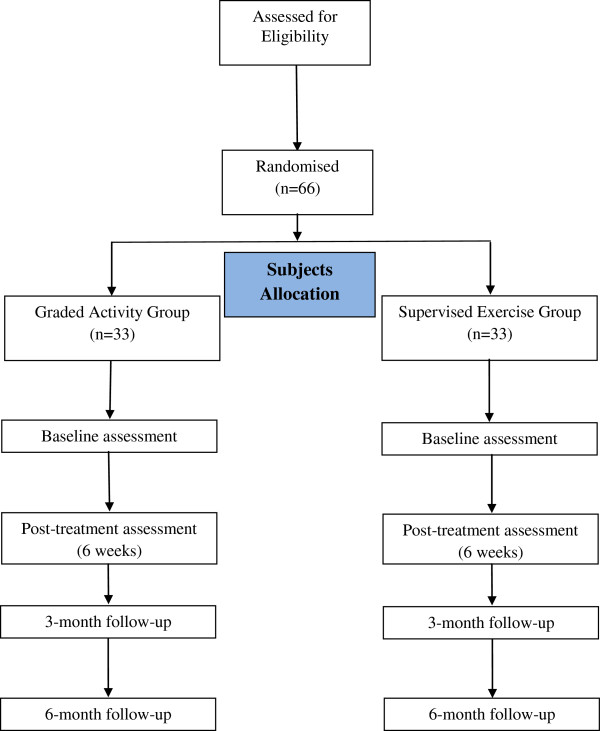
Flow diagram of the study.

Before treatment onset, the patients will be randomly allocated into one of two groups: Graded Activity (GA) or Supervised Exercise (SE), through a computer-generated randomisation schedule that will be performed by an independent researcher, not involved in other study procedures. The allocation of participants will be concealed by using consecutive numbered, sealed and opaque envelopes [31]. The flow of the study is summarized in Figure 1.

### Outcome measures

All instruments were used in their translated and adapted to Brazilian-Portuguese versions, with adequate psychometrical properties [24,25,32-34]. Pain, functional disability, return to work, physical activity, physical capacity and kinesiophobia will be measured.

### Primary outcomes

#### Pain

Pain will be assessed quantitatively and qualitatively with the *Numerical Rating Scale* (NRS) and the *McGill Pain Questionnaire*. The NRS is an 11-point scale ranging from 0 to 10, in which 0 defines absence of pain and 10 describes unbearable pain [24]. Participants will be asked to rate the average pain levels over the week before assessment [24].

The *McGill Pain Questionnaire* provides a multidimensional assessment of pain. It consists of 78 descriptors of the quantity and quality of pain which are grouped in four major domains (sensory, affective, evaluative and miscellanea) and 20 sub-domains with 1 to 5 descriptors each, to which intensity values are assigned. The questionnaire is used to describe pain experience and the score corresponds to the sum of the aggregated values. Maximal scores will be: Sensorial = 41, Affective = 14, Evaluative = 5, Miscellanea = 17, Total = 7 [35].

#### Functional disability

The *Roland Morris Disability Questionnaire* will be used to assess functional disability due to LBP. It consists of 24 questions focusing on normal activities of daily life. Each affirmative answer corresponds to 1 point and the final score is determined by the total number of points. Total score ranges from 0 to 24 and higher scores reflect increased disability. Scores above 14 reflect severe impairment [24,25,36].

### Secondary outcomes

#### Quality of life

*ShortForm Health Survey Questionnaire* (SF-36) assesses health-related qualify of life. It consists of 36 questions grouped in eight domains: vitality (4 items), Physical Functioning (10 items), Bodily Pain (2 items), General Health (5 items), Physical Role (2 items), Emotional Role (3 items), Social Functioning (2 items) and Mental Health (5 items). For each domain, scores range from 0 to 100 and higher scores reflect better quality of life. Only the physical and emotional domains will be used [32].

#### Global perceived effect

The *Global Perceived Effect Scale* is an 11-point scale that ranges from −5 (vastly worse), zero (no change) to +5 (completely recovered). For all of the measures of perceived global effect, the participants will be asked the following question: “Compared to when this episode first started, how would you describe your back?” Positive scores represent greater recovery and negative scores represent worsening of the symptoms [24].

#### Return to work

The evaluation will be made by questioning if the patient is off work due to back pain and the positive or negative response will be recorded.

#### Kinesiophobia

The *Tampa Scale of Kinesophobia* (TSK) is a self-applied questionnaire consisting of 17 items, which was developed to measure the fear of movement due to cLBP. Each question has 4 response options (strongly disagree, disagree, agree, and strongly agree) with scores respectively ranging from 1 to 4 points. The scores of items 4, 8, 12 and 16 are inverted and the total score is the sum of the items, which ranges from 17 to 68 points. Increased values reflect increased fear of movement [37,38].

#### Physical activity

The *Baecke Physicial Activity Questionnaire* measures physical activity in three domains: occupational activity, physical exercises and leisure/locomotion. It consists of 16 questions structured as a quantitative *Likert* scale. Its score is determined by the sum of the domain scores. Physical activity may be classified as mild (3.0-6.7), moderate (6.8-8.1) or intense (8.2-15.0) [34].

#### Physical capacity

The sit-to-stand and 15.24 m walk tests will be used. Five repetitions of the sit-to-stand test will be performed at maximal speed, without using the hands [39]. After five minutes of rest, the walk test will be performed, in which patients walk through 7.62 m, turn around and return to the initial position. The sit-to-stand and walk test will be assessed twice with an interval of three minutes, and the average value will be used for analyses [39]. Time will be measured using a digital manual stopwatch (instrutherm®).

#### 10-repetition maximum test (10-RM)

The 10-RM test measures the maximal load that allows participants to perform 10 complete repetitions. This test will be used for the flexor and extensor muscles of the knee. The progressive method will be used, beginning with smaller loads that will be progressively increased until participants can no longer generate the torque required for completing 10 repetitions. In-between each series, individuals will rest for 1 minute.

During the first two weeks of training, individuals will exercise using 50% of the maximum load. On the third and fourth week, the load will be 60% of maximum; for the final two weeks, it will be 70% [40].

### Intervention

Interventions will last 60 minutes and will happen twice a week for 6 weeks. They will be supervised by the investigator and participants will be asked to report any complaint (adverse events) related or not to treatment. They will also be instructed not to initiate any intervention while during the study. Ongoing medications will be maintained.

#### Supervised exercise program

Patients in the Supervised Exercise Group will perform stretching, strengthening and motor control exercises, as described in Table 1.

**Table 1:** Description of the protocol of the Supervised Exercise Group

**Exercise**	**Position**	**Sets/Duration**
Stretching	Stretching of the erector spinae in dorsal decubitus, with flexed hips and knees;	3 sets of 30 seconds
	Stretching of the hamstrings and triceps surae in dorsal decubitus, with forced flexion of 1 limb at a time with assistance of the physical therapist;	
	Stretching of the erector spinae with the patient sitting on heels, flexed trunk with the abdomen resting on the front of the thighs;	Intervals between series of 30 seconds
	Global stretching of the posterior muscular chain (erector spinae, hamstring, triceps surae.) 2 series of 4 minutes were performed, with 1 minute of resting interval.	
Strengthening	Exercises for the rectus abdominis in dorsal decubitus with flexed knees: trunk flexion;	2 sets of 12 repetitions
	Exercises for the rectus abdominis, external and internal obliquus in dorsal decubitus and flexed knees: trunk flexion and rotation;	
	Exercises for the rectus abdominis in dorsal decubitus and semi-flexed knees: hip flexion;	Intervals between series of 30 seconds
	Exercises for the erector spinae in ventral decubitus: trunk extension.	
Motor control	Exercises for the lumbar multifidus in ventral decubitus;	2 sets of 10 repetitions
	Exercises for the transversus abdominis muscle in dorsal decubitus with flexed knees;	
	Exercises for the transversus abdominis muscle in 4 point kneeling;	Intervals between series of 30 seconds
	Co-contraction of the transversus abdominis muscle and lumbar multifidus in the upright position.	

#### Graded activity

For this group, we will follow the protocols described by Macedo *et al.*[41] and Smeets *et al.*[42], which are based on individualized, progressive and sub-maximal exercises aiming to improve physical fitness and stimulate changes in behavior and attitudes due to pain. Positive reinforcement will be provided during the sessions (“you are doing great”, “congratulations”, “keep up with the good work”, “you can make it”), with the aim of maintaining the motivation.

In the beginning of the treatment, patients will select one or two activities considered difficult to them and receive guidance concerning them throughout the treatment, with the establishment of weekly goals. Participants will also receive an educational material (based on “Back Book”), with the purpose of providing important information about how to care for the spine. Weekly reading goals of the educational material will also be defined and the topics will be discussed at the end of each week. The protocol is described in Table 2.

**Table 2:** Description of the protocol of the Graded Activity Group

**Exercise**	**Position**	**Sets/Duration**
Aerobic training on the treadmill	5-minute warm-up with speed of 5–8 km/h;	
	20-minute submaximal training at 70-80% maximum heart rate;	
	5-minute slow-down with gradual speed reduction.	
Lower limbs strengthening	Exercise for the quadriceps in sitting position;	3 sets of 12 repetitions for each limb
	Exercise for the hamstrings in standing position.	Intervals between series of 30 seconds
Trunk strengthening	Exercises for the erector spinae in ventral decubitus: trunk extension.	3 sets of 10 repetitions
		Intervals between series of 30 seconds

Heart rate (HR) will be calculated using the formula of Karvonen (maximum HR = 200 – age) for sedentary individuals: Exercise HR = Resting HR + 70% to 80% of maximum HR [43].

### Sample size calculation

Sample size was defined in order to detect a 2-point difference between groups on the pain intensity outcome measured by the *Pain Numerical Rating Scale*, assuming a standard deviation of 1.9 points [24]. We also sought power to detect a 4-point difference in functional disability measured by the *Roland Morris Disability Questionnaire*, with an estimated standard deviation of 4.9 points [24,25,36]. Power was defined as 80% for an alpha of 5% and attrition (drop-outs) of 15%. Accordingly, 33 participants per group will be needed.

### Statistical analyses

All of the statistical analysis will be based on intention-to-treat basis [44]. We will use linear mixed models to test for treatment effects between groups at the end of the treatment and at three and six-month follow-up. Treatment effect sizes will be calculated for each time points, as well as statistical significance. Analyses will be conducted using SPSS 19 and SigmaPlot 10. A level of significance of 5% will be used.

### Ethics and data security

This trial was approved by the Ethics Committee of the School of Medicine of the University of Sao Paulo (protocol study-052826/2012). All patients will be asked to provide written, informed consent prior to randomisation, using standard forms. Data access and storage will be kept in accordance to the National Health and Medical Research Council guidelines. This trial is registered in ClinicalTrials.gov (a service of U.S. National Institutes of Health) under the number NCT01719276.

## Discussion

The purpose of this randomised controlled trial is to compare the effect of graded activity and supervised exercise in patients with non-specific cLBP. The study will contribute to clinical practice by providing evidence to guide decisions for the proper treatment of patients with cLBP. The results of this study will be published once the study is concluded.

## Competing interests

The authors declare that they have no competing interests.

## Authors’ contributions

MOM, FJR, LAVR, TNB, APMCCS, GPLA, SLKY were responsible for the design of the study. APM will act as the study coordinators. All authors read and approved the final manuscript.

## Pre-publication history

The pre-publication history for this paper can be accessed here:

http://www.biomedcentral.com/1471-2474/14/36/prepub
